# HSP70 as a Mediator of Host–Pathogen Interaction in *Arabidopsis thaliana* During *Plasmodiophora brassicae* Infection

**DOI:** 10.1111/ppl.70309

**Published:** 2025-06-04

**Authors:** Romana Kopecká, Miroslav Berka, Susann Auer, David Alabadí, Markéta Luklová, Sunita Jindal, Jutta Ludwig‐Müller, Martin Černý

**Affiliations:** ^1^ Department of Molecular Biology and Radiobiology Faculty of AgriSciences, Mendel University in Brno Brno Czech Republic; ^2^ Faculty of Biology, Department of Plant Physiology, Technische Universität Dresden Dresden Germany; ^3^ Instituto de Biología Molecular y Celular de Plantas (CSIC‐UPV) Valencia Spain

**Keywords:** clubroot disease, interactomics, plant immunity, plant‐pathogen interaction, proteomics

## Abstract

*Plasmodiophora brassicae* is one of the most devastating threats to Brassicaceae crops. However, the molecular mechanisms underlying clubroot disease remain unclear. Initial proteomics results led us to hypothesize that HSP70 proteins regulate host– *P. brassicae* interactions by modulating both plant defenses and pathogen activity. Using the *Arabidopsis thaliana* ‐ *P. brassicae* model system, we studied the role of HSP70 proteins in detail. Through a combination of proteomics and mutant phenotype analyses, we indicate that *Plasmodiophora* infection induces HSP70 accumulation in *Arabidopsis* roots, and mutations in specific HSP70 isoforms either promote (*HSP70‐1*, *HSP70‐13*, *HSP70‐14*) or suppress (*HSP70‐5*, *HSP70‐12*) the onset of clubroot disease. Proteomic profiling of root galls showed strong correlations between infection severity and pathogen‐derived HSP70 protein CEO96729. Interactomics analyses revealed that CEO96729 interacts with host proteins involved in plant response to *Plasmodiophora* infection, including an extracellular GDSL esterase/lipase with a putative role in long‐distance signaling, and that CEO96729 forms heterodimers with host HSP70 isoforms. These findings suggest that *Plasmodiophora* hijacks the host chaperone machinery to facilitate infection, offering a potential explanation for the observed modulation of disease progression in HSP70 mutants. Notably, the results also point to possible intracellular interactions with key enzymes in host physiology, including catalase 2, essential for ROS metabolism, and nitrilase, critical for auxin biosynthesis and root gall formation. Collectively, our study highlights the multifaceted roles of HSP70 proteins in *Plasmodiophora* pathogenicity and host–pathogen interactions, providing insights into chaperone‐mediated processes in plant immunity and infection dynamics.

## 1 Introduction

Clubroot disease, caused by the pathogen *Plasmodiophora brassicae* , represents one of the most devastating threats to Brassicaceae crops, resulting in significant global yield losses (Dixon 2009). Over the past decade, its geographic prevalence has expanded alarmingly, with confirmed cases increasing from 60 to over 80 countries, affecting all continents except Antarctica (Hwang et al. 2012; Javed et al. 2023). A recent study of *P. brassicae* samples collected from oilseed rape ( *Brassica napus* ) fields across Europe revealed an increase in the virulence of naturally occurring pathotype populations over 5 years (Zamani‐Noor et al. 2022). As of 2020, clubroot incidence, that is, the number of plants that were infected, reached up to 80% in sampled fields in Germany and up to 27% in the Czech Republic. The prevalence of evolving pathotypes capable of overcoming clubroot resistance (CR) also appears to be increasing in these regions (Řičařová et al. 2016; Zamani‐Noor et al. 2022).

*P. brassicae* is a soil‐borne, obligate biotrophic protist pathogen with a complex life cycle that includes primary and secondary infection stages (Pérez‐López et al. 2018). The primary infection targets root hairs, while the secondary infection occurs within the root cortex, leading to gall formation, impaired nutrient uptake, and ultimately plant death (Kageyama and Asano 2009). Current control strategies, such as crop rotation, soil liming, and the use of resistant cultivars, often prove inadequate due to the remarkable persistence of *P. brassicae* resting spores in soil and the emergence of new virulent pathotypes (Botero‐Ramírez et al. 2022; Struck et al. 2022). Clubroot‐resistant varieties containing a single *CR* gene, such as those derived from the cultivar “Mendel,” are not cultivated as extensively in Europe compared to regions like Canada, where pathogen populations are known to evolve rapidly under the selection pressure imposed by short rotations of the same resistant cultivars (LeBoldus et al. 2012; Cao et al. 2014). Nevertheless, between one‐third and two‐thirds of *P. brassicae* isolates from Central European soils were able to overcome the Mendel‐derived resistance in *B. napus* , and a smaller proportion were capable of overcoming additional resistance sources from other Brassica species (Zamani‐Noor et al. 2022). Addressing this challenge necessitates innovative agricultural solutions grounded in a deeper understanding of the molecular mechanisms governing *P. brassicae* infection and host‐pathogen interactions.

A crucial aspect of understanding the infection process involves identifying pathogen effector proteins (Pérez‐López et al. 2018). A major advancement in understanding *P. brassicae* pathogenicity was the discovery of a methyltransferase that suppresses salicylic acid defenses by converting it into inactive methyl salicylate (Ludwig‐Müller et al. 2015). Salicylic acid mitigates clubroot severity in Brassica species by enhancing antioxidant enzymes, regulating osmotic stress, modulating reactive oxygen species (ROS), and triggering immune responses (Galindo‐González et al. 2020; Ji et al. 2021; Xi et al. 2021; Wang et al. 2022). Interestingly, plant hormone balance seems to play a central role in *P. brassicae* interaction (Jayasinghege et al. 2023). *P. brassicae* upregulates jasmonic acid‐related genes in *Arabidopsis thaliana* , and a suppression of its pathway promotes gall development (Irani et al. 2018; Wang et al. 2022). Ethylene influences resistance by activating salicylic acid signaling early in the infection but may promote susceptibility later by inhibiting jasmonic acid signaling (Galindo‐González et al. 2020; Wang et al. 2022). The process of gall formation itself is governed by cytokinin and auxin (Prerostova et al. 2018; Bíbová et al. 2023).

Proteomic studies have contributed to revealing the complex interplay between *P. brassicae* and its hosts. The identified processes include common biotic interaction pathways, such as oxidative stress response, calcium signaling, phytohormone metabolism, carbohydrate metabolism, and cell wall metabolism (Cao et al. 2008; Moon et al. 2020; Stefanowicz et al. 2021; Adhikary et al. 2022). These studies also pinpointed putative targets for promoting resistance, including S‐adenosylmethionine synthetase and glutathione S‐transferase (Ji et al. 2018; Lan et al. 2019). However, standard bottom‐up proteomics faces several challenges when analyzing biotic interactions. For instance, a substantial proportion of peptides are evolutionarily conserved, making it difficult to accurately distinguish between those originating from the microbe and those derived from the host (Berka et al. 2020). The second issue is a well‐known limitation of conventional proteomics workflows. Many experiments underestimate changes in highly abundant proteins, as these often fail to meet commonly accepted fold change thresholds and are consequently excluded from the list of differentially abundant candidates. Furthermore, accurately identifying and quantifying proteins belonging to large families with shared tryptic peptides remains a significant challenge. All three of these limitations are exemplified by the HSP70 protein family, which is highly evolutionarily conserved, includes members typically present at high abundance, and has recently gained broader attention in plant–microbe interaction studies. Here, we combine proteome profiling of the early response to *P. brassicae* in *Arabidopsis thaliana* with in‐depth proteome analyses of developing root galls in both wild‐type and mutant *Arabidopsis* plants. Our findings strongly suggest that HSP70 proteins are key players in the plant's response to *P. brassicae* . Furthermore, our protein–protein interaction analyses reveal that *P. brassicae* HSP70 readily forms heterodimers with its host counterparts. This interaction suggests potential mechanisms by which the pathogen interferes with and/or exploits the host's metabolism.

## 2 Materials and Methods

### 2.1 Plant Material

The majority of the reported experiments are based on the model plant *Arabidopsis thaliana* (Col‐0) and its derived mutants. Seeds of *Arabidopsis thaliana* Columbia‐0 (Col‐0), *hsp70‐1* (SALK_135531C), *hsp70‐2* (SALK_085076.51.65.x), *hsp70‐5* (SAIL_839_A08C1), *hsp70‐12* (SALK_047956C), *hsp70‐13* (GABI_075D06), *hsp70‐14* (SALK_082815.45.00.x), and *hsp70‐16* (SALK_125951.45.30.x) were surface‐sterilized with 70% ethanol and sown on half‐strength Murashige and Skoog medium solidified with 1.2% agar. Seeds were stratified at 4°C for 3 days and then transferred to growth chambers under controlled conditions (21°C, 12‐h photoperiod, and 100 μmol m^−2^ s^−1^ of photon flux density). After 10 days, plants were transplanted into sterilized soil and grown in a greenhouse under a 12‐h photoperiod. For capturing protein–protein interactions, *Nicotiana benthamiana* (a commonly used model for transient transformations) and *Brassica napus* (cv. Sázava, a close relative of *Arabidopsis* and a natural host of *P. brassicae* ) seeds were sown directly into soil and grown in growth chambers under controlled conditions: 21°C, 12‐h photoperiod, and 100 μmol m^−2^ s^−1^ (*N. benthamiana*) or 300 μmol m^−2^ s^−1^ ( *B. napus* ) photon flux density for up to 6 weeks. Similar conditions were used for cultivating *B. napus* for xylem sap analyses, with plants preincubated with flg22, a conserved peptide sequence derived from bacterial flagellin (QRLSTGSRINSAKDDAAGLQIA, > 95% purity; ProteoGenix). The flg22 solution (1 μM flg22, 0.025% v/v Silwet L‐77) was applied by spraying the leaves and pouring the solution under the pot. Plants received 30 mL for spraying and 200 mL for watering.

### 2.2 Plant Inoculation and Disease Assessment

Twelve‐day‐old *Arabidopsis thaliana* plants were inoculated with *P. brassicae* isolate ‘e3’ (Fähling et al. 2003; 10^6^–10^7^ spores/mL in 50 mM KH_2_SO_4_, pH 5.5). Two milliliters of spore suspension were pipetted onto the soil surrounding each plant. Disease severity was assessed 28 days post‐inoculation by rating the plants on a scale of 0–4 and calculating a disease index (DI, Siemens et al. 2002); scale of 0–4: 0 = no symptoms; 1 = minor swellings; 2 = thickened primary and lateral roots; 3 = reduced root system with visible galls; 4 = single, large gall. DI experiments are based on at least 25 plants, each inoculated independently, with every plant receiving the same amount of inoculum from the same spore batch. In parallel, mutant lines were evaluated using relative growth rate (RGR) at 13, 16, and 19 days post‐inoculation. RGR was calculated as the natural logarithm of the apparent rosette area at each time point minus the natural logarithm of the initial (previous) apparent rosette area, divided by the time interval between the two measurements.

### 2.3 Yeast Two‐Hybrid Screening

A yeast two‐hybrid screen was performed using the CEO96729 gene cloned into the bait vector pGBKT7. The Matchmaker Gold Yeast Two‐Hybrid System and a universal Arabidopsis cDNA library (Takara, Cat. No. 630487) were employed according to the manufacturer's protocol. Positive colonies were selected on DDO/X/A medium and subsequently screened on more stringent QDO/X/A medium. Plasmids from positive colonies were isolated using an Easy Yeast Plasmid Isolation Kit (Takara Bio) and transformed into *E. coli* DH5α. Plasmid DNA was isolated and sequenced to identify interacting proteins (SEQme s.r.o.).

### 2.4 Transient Expression of HSP70 and *ce* 096729

The *HSP70‐1* and *ce096729* genes were cloned into expression vectors using Gateway and GreenGate cloning systems, respectively. For *ce096729*, the coding sequence (CDS) was amplified by PCR and cloned into the pDONR207 entry vector. Subsequently, the CDS was transferred to the pEarleyGate104 destination vector to introduce an N‐terminal YFP tag. For *HSP70‐1*, the CDS was amplified and cloned into the pGGC000 vector. The GFP tag and linker were cloned into pGGD000 and pGGF000, respectively. The final construct with a C‐terminal GFP tag was assembled using the GreenGate protocol and the pFASTRK destination vector (Decaestecker et al. 2019). For details, see Figure S1.

*Agrobacterium tumefaciens* GV300 cells were transformed with three expression constructs (*p35S:YFP:CEO96729:tOCS*, *p35S:HSP70‐1:GFP:tRBCS*, *p35S:GFP:tRBCS*) and grown in LB medium supplemented with rifampicin, gentamicin, and kanamycin/spectinomycin at 28°C. Bacterial cultures were adjusted to an OD_600_ of 0.5, pelleted, and resuspended in infiltration buffer (10 mM MgCl_2_, 200 μM acetosyringone, 10 mM MES pH 5.6). After a 2‐h incubation in the dark, the bacterial suspensions were infiltrated into leaves of six‐week‐old *N. benthamiana* or *B. napus* plants.

### 2.5 Confocal Microscopy

Two days post‐infiltration, YFP and GFP fluorescence were visualized using Zeiss LSM 700 and LSM 5 Pascal confocal microscopes. Fluorescence signals were detected in the 510–560 nm emission range.

### 2.6 Protein Sample Preparation

Total protein extracts were isolated from ground shoot and root tissues using a previously described acetone/TCA/phenol extraction method (Dufková et al. 2023). Early response proteome analysis was performed using three biological replicates. Root gall development in mutant lines was investigated across four biological replicates (category 4) and modeled using at least two biological replicates per category. Proteome dynamics in Col‐0 gall roots were analyzed using up to nine biological replicates per category. All plants in the disease ranking experiment were analyzed (categories 0, 1: *n* = 5; category 3, 4: *n* = 9). Xylem sap proteome was extracted and analyzed, as described previously, using five biological replicates (Kopecká and Černý 2024).

### 2.7 Proteome Analyses—Co‐Immunoprecipitation

Leaves of transiently transformed *N. benthamiana* and *B. napus* plants were harvested, flash‐frozen, and lyophilized. Approximately 80 mg (dry weight) of leaf tissue was homogenized using a Retsch mill and extracted on ice with 1 mL of native extraction buffer (150 mM NaCl, 0.5% NP‐40, 1 mM EDTA, 150 mM Tris–HCl pH 8.0) supplemented with a plant protease inhibitor cocktail (Sigma, 40 μL mL^−1^). Protein extracts were then clarified by centrifugation (10,000 *g* for 10 min at 4°C), and GFP‐tagged HSP70 proteins and their interacting partners were immunoprecipitated using 25 μL of GFP‐Trap Magnetic Agarose beads (ChromoTek, Planegg‐Martinsried, Germany) according to the manufacturer's protocol. Briefly, the sample with beads was incubated on a rotator for 60 min at 4°C. Beads with bound proteins were collected by centrifugation (2500 *g*, 4°C), washed twice with 500 μL of extraction buffer, and captured proteins were eluted with 50 μL of 0.2 M triethylamine. The eluate was immediately neutralized with 1 M MES and mixed with 300 μL of solubilization buffer (8 M urea, 50 mM ammonium bicarbonate). Protein samples were then reduced, alkylated, and digested with trypsin (0.5 μg per sample). The experiments primarily targeted *Plasmodiophora* HSP70 interactors, with six replicates in *N. benthamiana* and eight in *B. napus* (one replicate was lost). The GFP and Arabidopsis HSP70 constructs were used as independent controls to account for nonspecific interactions and identify HSP70‐specific targets, respectively. These experiments were conducted with four biological replicates in *B. napus* and three in *N. benthamiana*.

### 2.8 Liquid Chromatography Coupled With Mass Spectrometry (LC–MS)

For initial proteomic analysis, a gel‐free shotgun approach was employed. Samples (5 μg peptide based on Bradford assay protein content estimation prior to digestion) were separated using nano‐HPLC on a 15 cm C18 Zorbax column and analyzed by MS/MS on a UHR maXis impact q‐TOF mass spectrometer (Bruker). A third biological replicate was validated using a TSQ Quantiva Triple‐stage Quadrupole mass spectrometer (Thermo Fisher Scientific) (Dufková et al. 2019, 2023). Root gall proteomes and co‐immunoprecipitated samples were characterized using an Orbitrap Fusion Lumos Tribrid Mass Spectrometer as detailed in Berková et al. 2023. In all experiments, an equivalent of 5 μg of peptides was injected for LC–MS analysis, based on protein content estimation before digestion or total ion current (for xylem sap proteome and co‐immunoprecipitation experiments).

### 2.9 Proteome Data Analysis

The measured spectra were recalibrated and searched against the Araport 11 ( *A. thaliana* ), *Plasmodiophora brassicae* (GCA_001049375.1), *B. napus* 2.0 (PRJNA237736), *N. benthamiana* (Kourelis et al. 2018) and common contaminants databases using Proteome Discoverer 2.5 (Thermo Fisher Scientific) employing Sequest HT, MS Amanda 2.0 (Dorfer et al. 2021), or MSFragger algorithms (Kong et al. 2017). The quantitative analysis centered on (1) proteins identified by two or more unique peptides and (2) proteins with a single unique peptide but at least 10 assigned peptides, aiming for broader proteome coverage. The mass spectrometric proteomic data acquired were deposited in the ProteomeXchange Consortium (http://proteomecentral.proteomexchange.org) via the PRIDE partner repository (Perez‐Riverol et al. 2022) with the dataset identifier PXD059131.

### 2.10 Bioinformatic Analyses and Statistics

Protein structures CEO96729 (GMQE 0.72) and HSP70‐1 (GMQE 0.89) were aligned using SWISS‐MODEL (Guex et al. 2009; swissmodel.expasy.org). Conserved motifs were identified with MEME Suite (Bailey et al. 2015; http://meme‐suite.org/tools/meme). Phylogenetic trees were constructed using ETE3 (Huerta‐Cepas et al. 2016) as implemented on GenomeNet (https://www.genome.jp/tools‐bin/ete), followed by midpoint rooting and visualization with iTOL (Letunic and Bork 2024, https://itol.embl.de/). Structure prediction was performed using AlphaFold2 through Neurosnap (Neurosnap Inc., 2022; https://neurosnap.ai/) and visualized with Mol* Viewer (Sehnal et al. 2021). Significant differences refer to *p* < 0.05; adj. *p* value represents Benjamini and Hochberg procedure at 5% FDR. In brief, statistical analyses included Student's *t*‐test followed by FDR correction for pairwise comparisons (e.g., *P. brassicae* vs. mock in early response proteomics, individual *hsp70*s vs. Col‐0 in galls category 4, comparisons of individual categories 1–4 vs. category 0, and the comparison of CEO96729 and HSP70‐1 interactors). The Kruskal–Wallis test, followed by Conover's post hoc test, was used to analyze HSP70 abundances in mutants, evaluate disease progression, assess RGRs, and compare pathogen load. An orthogonal partial least squares model and the variable importance in projection were employed to model the proteome response to pathogen load. The corresponding results are presented in the figures and the Supporting Information tables. Statistical tests were performed using established software packages, including Real Statistics Resource Pack (Release 6.8; Copyright 2013–2020; Charles Zaiontz; www.real‐statistics.com), SIMCA 14.1 (Sartorius), and Proteome Discoverer 2.5 (Thermo Fisher Scientific).

## 3 Results

### 3.1 *P. brassicae* Induces the Accumulation of HSP70 in *Arabidopsis thaliana*

**FIGURE 1:**
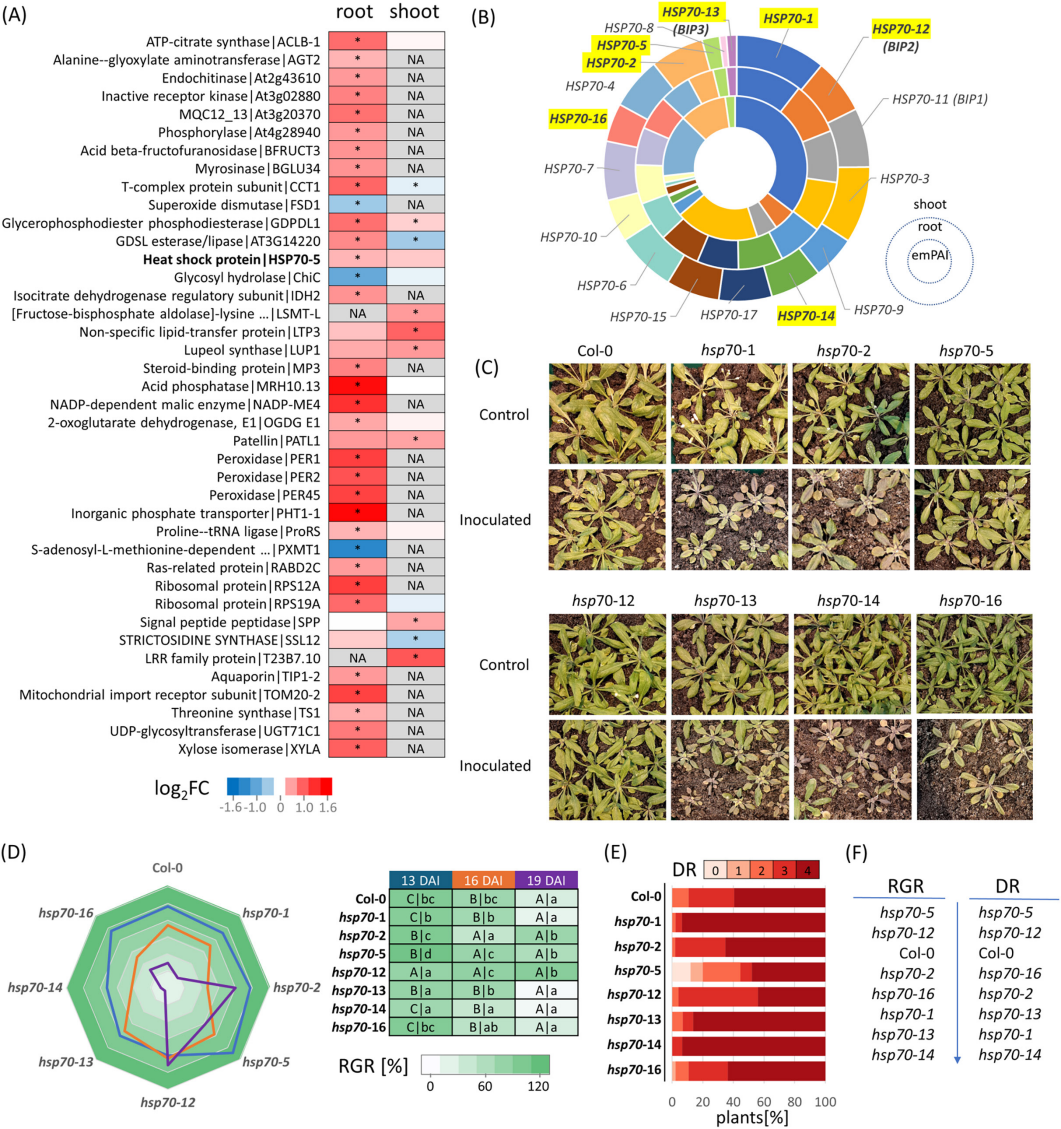
HSP70 has a role in *Plasmodiophora brassicae* infection. (A) Differentially abundant proteins identified in the roots and shoots of 4‐week‐old *A. thaliana* Col‐0 plants collected 14 days after inoculation (DAI). Asterisks indicate significant differences compared to mock‐inoculated plants (*p* < 0.05, relative fold change > 1.4). Based on three biological replicates, each consisting of a pool from at least five individual plants; (B) expected abundance of HSP70s in the root and shoot of *Arabidopsis*. Mean expression values of all *HSP70* members in *Arabidopsis* leaf (outermost shell) and root (intermediate shell) tissues based on the ATHENA database (O'Connor et al. 2005), and estimated root protein content (innermost shell) in roots collected 14 DAI based on emPAI. HSP70 members in bold indicate candidates used for mutant experiment; (C) impact of *HSP70* mutation on clubroot disease onset. Representative images of plants at 19 DAI; (D) comparison of relative growth rates (RGRs). RGR ratios (inoculated:Control) were calculated at 13, 16, and 19 DAI. Data are based on the apparent leaf area of at least 20 plants. Statistical significance was determined using the Kruskal–Wallis and Conover tests (*p* < 0.05). Capital and lowercase letters indicate within‐ and between‐line comparisons, respectively; (E) disease rating (DR) based on root gall development (0—healthy, 4—largest galls developed. For representative images of galls, see Figure S2; (F) genotype resilience rating based on RGR and DR rankings.

Our initial proteomic analysis identified a subset of *Plasmodiophora brassicae* ‐responsive proteins in *Arabidopsis thaliana* Col‐0. Shoots and roots of four‐week‐old plants were harvested 14 days post‐inoculation (DPI), capturing the critical time point prior to the onset of visible disease symptoms. A combined analysis of root and shoot proteomes revealed 3069 unique proteins, of which 33 exhibited significant differences in abundance within the root tissue (comparison with mock‐inoculated plants, *p* < 0.05; relative fold change > 1.4; Figure 1A, Table S1). Notably, only three proteins were significantly less abundant, including superoxide dismutase SOD1. Among the accumulated proteins, we identified several proteins of interest, such as NADP‐dependent malic enzyme 4, peroxidases, GDSL esterase/lipase, endochitinase, aquaporin TIP1‐2, threonine synthase, and heat shock protein 70‐5 (HSP70‐5). The latter drew particular attention as HSP70s have been previously shown to play pivotal roles in diverse plant–microbe interactions (Berka et al. 2022). Interestingly, based on publicly available expression data, HSP70‐5 was found to have relatively low basal expression levels (Figure 1B). This was corroborated by our proteomic analysis, where exponentially modified protein abundance index (emPAI) estimated HSP70‐5 abundance to be approximately 2% of the total HSP70 family protein content (Figure 1B).

### 3.2 HSP70 Modulates *Arabidopsis thaliana* ‐ *P. brassicae* Interactions

To validate the role of HSP70 in the *P. brassicae* response, we assessed the response to *P. brassicae* inoculation in the *hsp70‐5* mutant line. To expand our investigation, we also examined mutants in the presumably most abundant root isoforms, *HSP70‐1* and *HSP70‐12*, as well as candidate HSP70s predicted to localize to the cytosol (*HSP70‐2*, *14*, and *16*) and the endoplasmic reticulum (ER) stress‐response protein *HSP70‐13* (BIP3), which has been implicated in innate immunity (Park et al. 2010). Mutation of *HSP70* genes significantly impacted plant resilience to *P. brassicae* . Two mutants, *hsp70‐5* and *hsp70‐12*, exhibited inhibited or delayed disease onset. However, most of the analyzed mutants displayed more pronounced disease symptoms than the wild‐type Col‐0. The RGR, based on apparent leaf area, was significantly lower in all inoculated mutants except *hsp70‐12*. While *hsp70‐5* and *hsp70‐12* showed a lower or no decrease in RGR compared to Col‐0, all other mutants exhibited a more rapid decline in RGR (Figure 1C,D). At 28 DAI, plants were uprooted, and root tissue was assessed and ranked based on disease symptom development (Figure 1E). Among all genotypes, *hsp70‐5* stood out as the only mutant displaying resistance in a subset of individuals, but enhanced resilience was also confirmed for *hsp70‐12*. A comparison of rankings based on mean RGR and disease rating revealed a strong positive correlation (Pearson's *r* = 0.92, *p* < 0.01), with both *hsp70‐5* and *hsp70‐12* ranking above the wild‐type Col‐0, and the remaining mutant lines exhibiting more severe symptoms. Interestingly, *hsp70‐1* had the highest percentage of individuals in the most severe disease category (category four).

### 3.3 Root Gall Proteomes Showed Similarities in the Gall Composition and Estimated Pathogen Load

**FIGURE 2:**
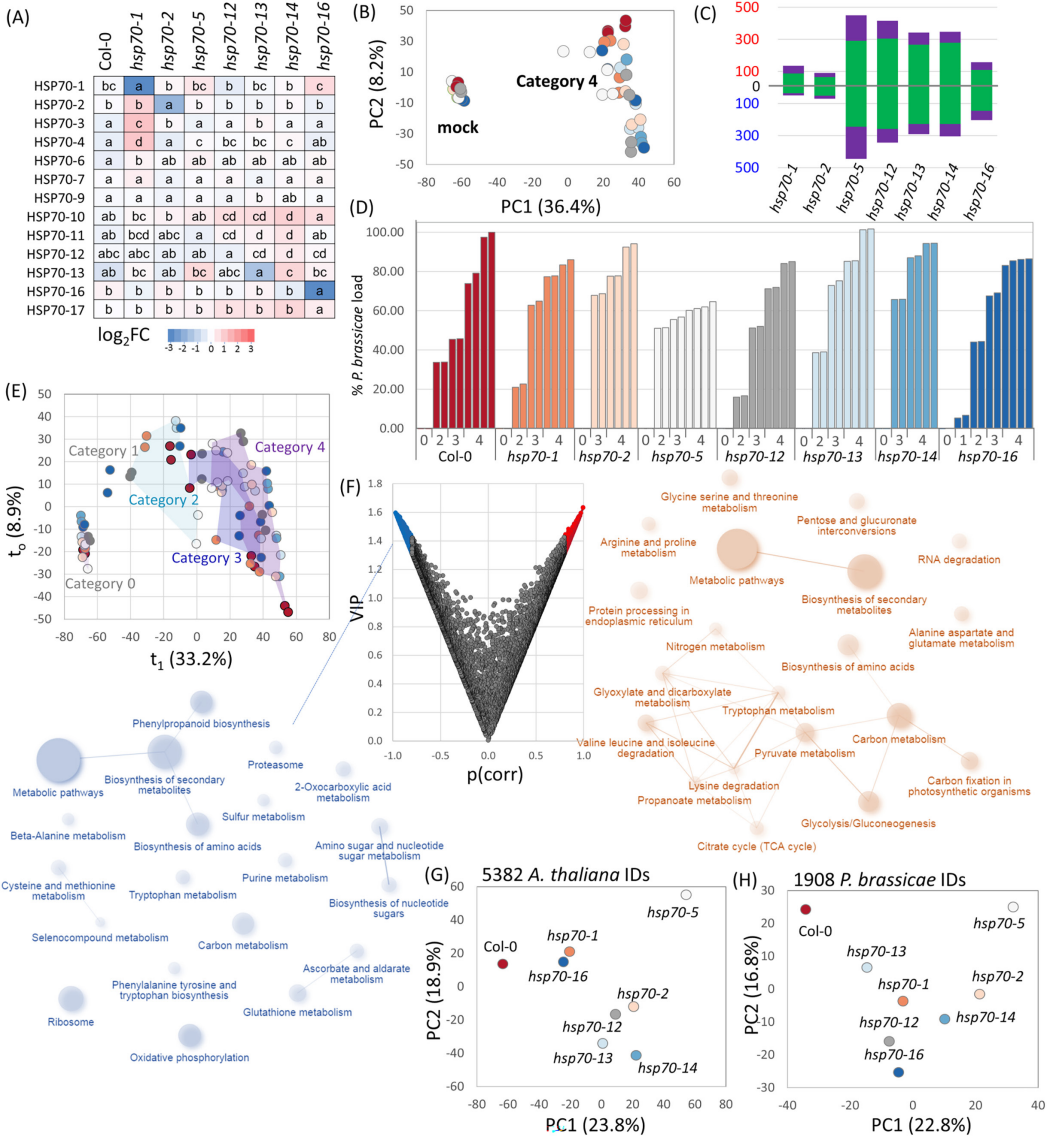
Mutations in *HSP70* modulate root gall proteomes. (A) Estimated abundances of identified HSP70 proteins in control root tissue. Results based on the manual inspection of extracted chromatograms. Statistical significance was determined using the Kruskal–Wallis and Conover tests (*p* < 0.05). For heatmap visualization, protein abundance values were normalized by the median abundance of each protein across all samples; (B) separation of proteome profiles based on identified *A. thaliana* proteins; (C) differentially abundant proteins in fully developed galls of mutant lines (DR = 4) compared to Col‐0 (adj. *p* < 0.05, four biological replicates). Green, *A. thaliana* proteins; purple, *P. brassicae* proteins; (D) estimated *P. brassicae* load based on summed abundances of identified *P. brassicae* proteins. Only proteins exquisitely found in infected tissues were used for the calculation. The numbers under bars correspond to the DR categories (0 = no symptoms; 1 = minor swellings; 2 = thickened primary and lateral roots; 3 = reduced root system with visible galls; 4 = single, large gall). Relative load values were normalized to the maximum load observed in Col‐0 galls of category 4; (E) orthogonal partial least‐squares discriminant analysis of proteome profiles based on estimated *P. brassicae* abundances followed by (F) VIP (variable importance in projection) and enrichment analysis of *Arabidopsis* proteins whose abundances negatively (blue) and positively (red) correlate with *P. brassicae* infection. The VIP score reflects the importance of each protein in explaining the variance in the model. Higher VIP scores indicate proteins that contribute more significantly to predicting the severity of *P. brassicae* infection. The p(corr) values represent the Pearson correlation coefficient between each protein's abundance and the OPLS model's first component, which is associated with pathogen load. Enrichment analysis is based on hypergeometric distribution followed by FDR correction. Two pathways are connected if they share 20% or more proteins. Darker and bigger nodes are more significantly enriched and larger sets, respectively; (G, H) comparison of proteome changes in the course of *P. brassicae* infection in *Arabidopsis* (G) and *P. brassicae* (H) proteomes based on the VIP projections. *P. brassicae* proteins detected in mock‐treated samples were identified as potential false positives and subsequently excluded from the final analysis. The color coding of mutant lines in the PCA (B, G, H) and OPLS (E) plots is consistent and corresponds to the colors shown in plot (D). Root gall development in mutant lines was investigated across four biological replicates (category 4) and modeled using at least two biological replicates per category. For details, see Tables S2–S4.

Despite the enhanced resistance observed in *hsp70‐5* and *hsp70‐12* mutants, all mutant genotypes eventually developed galls, suggesting that while an *HSP70* mutation can interfere with the pathogen, *P. brassicae* ultimately overcomes this inhibition. To gain deeper insights into the underlying mechanisms, we collected roots and galls from infected plants, categorized root galls into four categories based on clubroot development, and compared their proteome profiles (Figure 2A–H). In total, the analysis yielded reliable quantification of 5396 *Arabidopsis* and 2271 *P. brassicae* proteins (Table S2). The comparison of mock and category four proteomes separated infected and control samples (Figure 2B). Pairwise comparisons with Col‐0 galls highlighted similarities between Col‐0 and both *hsp70‐1* and *hsp70‐2*. The highest number of differentially abundant proteins was found in *hsp70‐5* and *hsp70‐12*, aligning with their observed differences in resilience (Figure 2C). Based on estimated protein abundances, *P. brassicae* contributed significantly to the extracted proteome, exceeding 30% estimated protein content in the late categories of root gall development. By leveraging proteins uniquely present in infected tissues, we estimated relative pathogen load, which correlated well with the classification of root galls (Figure 2D). This estimate was used to construct an orthogonal partial least squares (OPLS) model (Figure 2E). The variable importance in projection (VIP) scores (Figure 2F) identified *Arabidopsis* proteins positively (378 proteins) and negatively (569 proteins) correlated with pathogen load (Table S3). Both sets of proteins exhibited a wide range of metabolic pathway enrichments, indicating significant metabolic shifts. Host proteins increasing with pathogen load were primarily involved in catabolic processes, amino acid and RNA degradation, and energy production. Conversely, the decrease in abundance of proteins was associated with anabolic processes, including amino acid and nucleotide biosynthesis, secondary metabolite production, and glutathione metabolism. Intriguingly, the *P. brassicae* proteome also displayed gradual metabolic adjustments. While the evaluation was limited by insufficient annotation, *P. brassicae* proteins increasing in abundance with pathogen load were enriched in response to oxidative stress (GO:0006979), RNA splicing, and mRNA processing (GO:0008380, GO:0006397), and energy metabolism. A decrease was observed for carboxylic acid metabolism (GO:0019752) and primary metabolic processes (GO:0044238), potentially coinciding with the exploitation of host metabolism. Finally, the dynamics of *Arabidopsis* and *P. brassicae* protein abundances across gall development were visualized and compared across all lines (Figure 2G,H). The projections are based on individual OPLS/VIP models for each analyzed line, with each model identifying proteins that positively or negatively correlate with pathogen load. Complete results are available in Table S3. In both PCA projections, *hsp70‐5* showed the most divergent profile relative to Col‐0.

### 3.4 Possible Candidate Proteins Contributing to Altered Resistance in *hsp70* Mutants

Although a subset of individuals in all mutant lines remained susceptible to the pathogen, modeling revealed that the correlation between protein abundance and pathogen load differed between genotypes. Specifically, the comparison of *hsp70‐1* and *hsp70‐5* identified over 80 proteins that showed strong correlations with pathogen load in both genotypes (|r| > 0.5), but in opposite directions—positive in one and negative in the other (Table S3). Of these, 41 exhibited profiles similar to *hsp70‐1* in *hsp70‐14*, supporting their potential involvement in the enhanced resistance observed in the *hsp70‐5* genotype (Table 1). A substantial proportion of these proteins were of *Plasmodiophora* origin, suggesting that the pathogen may have adapted to the altered HSP70 pool in the host. Among the *Arabidopsis* proteins less abundant in the *hsp70‐5* were enzymes involved in amino acid catabolism (methylcrotonoyl‐CoA carboxylase subunit), a pre‐mRNA splicing factor, a steryl glycoside synthase, and a heat‐specific cofactor essential for acclimation and thermal stress survival. Conversely, proteins that accumulated in *hsp70‐5* included catalase 2 (involved in ROS metabolism), 4‐coumarate—CoA ligase (a key enzyme in the phenylpropanoid pathway), glutathione S‐transferase U6 (detoxification), and several enzymes linked to lipid metabolism, potentially affecting both membrane integrity and oxylipin signaling. Together, these proteins could be involved in multiple facets of plant defense, including signal transduction, transcriptional regulation, detoxification of reactive species, and reinforcement of structural barriers. Their differential abundance may help explain the enhanced resilience of the *hsp70‐5* genotype.

**TABLE 1:** Candidate proteins potentially contributing to altered resistance in *hsp70* mutants. Arrows indicate positive (↑, *r* > 0.5) and negative (↓, r < −0.5) correlations between protein abundance and pathogen load as identified in the OPLS model. For details, see Table S3.

*hsp70‐1*	*hsp70‐14*	*hsp70‐5*	Protein
↑	↑	↓	AT1G36160|Acetyl‐CoA carboxylase 1
↑	↑	↓	AT3G15450|DUF3700 domain‐containing protein
↑	↑	↓	AT5G21160|La‐related protein 1A
↑	↑	↓	AT1G03090|Methylcrotonoyl‐CoA carboxylase
↑	↑	↓	AT4G38780|Pre‐mRNA‐processing‐splicing factor 8B
↑	↑	↓	AT3G07020|Sterol 3‐beta‐glucosyltransferase UGT80A2
↑	↑	↓	AT2G39280|Ypt/Rab‐GAP domain
↓	↓	↑	AT1G24360|3‐oxoacyl‐[acyl‐carrier‐protein] reductase, chloroplastic
↓	↓	↑	AT3G21230|4‐coumarate‐CoA ligase 4
↓	↓	↑	AT5G65110|Acyl‐coenzyme A oxidase 2
↓	↓	↑	AT4G35090|Catalase‐2
↓	↓	↑	AT2G29440|Glutathione S‐transferase U6
↓	↓	↑	AT1G35780|N‐lysine methyltransferase
↓	↓	↑	AT1G01940|Peptidyl‐prolyl cis‐trans isomerase CYP18‐1
↓	↓	↑	AT1G03280|Transcription factor TFIIE
↓	↓	↑	AT3G62830|UDP‐glucuronic acid decarboxylase 2
↑	↑	↓	CEO96057|AAA+ ATPase domain‐containing protein
↑	↑	↓	CEP01523|Activator of Hsp90 ATPase homologue
↑	↑	↓	CEO97809|Asparagine‐tRNA ligase
↑	↑	↓	CEP02030|DUF5678 domain‐containing protein
↑	↑	↓	CEO98624|Exonuclease VII large subunit
↑	↑	↓	CEO99068|Fe2OG dioxygenase domain‐containing protein
↑	↑	↓	CEO97733|Hemerythrin‐like domain‐containing protein
↑	↑	↓	CEP00725|KOW domain‐containing protein
↑	↑	↓	CEP03315|Mediator of RNA polymerase II transcription subunit 11
↑	↑	↓	CEO94481|NTF2 domain‐containing protein
↑	↑	↓	CEO94463|O‐methyltransferase domain‐containing protein
↑	↑	↓	CEO97912|Peptidase C1A papain C‐terminal domain‐containing protein
↑	↑	↓	CEO97960|Phosphoenolpyruvate carboxykinase (GTP)
↑	↑	↓	CEO97625|Thioredoxin domain‐containing protein
↑	↑	↓	CEO95738|TRAF‐type domain‐containing protein
↑	↑	↓	CEO95025|Uncharacterized protein
↑	↑	↓	CEO97240|Uncharacterized protein
↑	↑	↓	CEO97873|Uncharacterized protein
↑	↑	↓	CEP01453|Uncharacterized protein
↑	↑	↓	CEP01640|Uncharacterized protein
↑	↑	↓	CEP03547|UspA domain‐containing protein
↓	↓	↑	CEO95534|3‐deoxy‐7‐phosphoheptulonate synthase
↓	↓	↑	CEP01682|Cullin family profile domain‐containing protein
↓	↓	↑	CEO99045|Mannose‐6‐phosphate isomerase
↓	↓	↑	CEO98230|Nucleolar protein 56

### 3.5 Mutation in *HSP70* Triggers Compensatory Accumulation of Other HSP70 Isoforms

The *Arabidopsis thaliana* HSP70 family comprises 18 members, suggesting functional redundancy among isoforms. To investigate this, we analyzed the root proteomes of the *hsp70* mutant lines compared to the wild‐type Col‐0. As anticipated, the abundance of the targeted HSP70 isoforms was significantly reduced or completely absent in the corresponding mutants, and their absence triggered compensatory increases in the levels of other HSP70 isoforms, revealing intricate regulatory dynamics within the HSP70 family. For example, the mutation in *HSP70‐1* led to a significant increase in the abundance of isoforms 3, 4, and 6 (Figure 2A, Table S4). While HSP70‐5 and HSP70‐14 were below the detection limit in wild‐type roots, their mutation impacted the abundance of other HSP70 isoforms. Notably, the mutation of HSP70‐5 resulted in an increase in HSP70‐1 levels (1.5‐fold increase compared to Col‐0, Student's *t*‐test *p* < 0.05, estimated abundance based on three unique peptides).

### 3.6 HSP70 Proteins: A Duel Between Host and Pathogen in *P. brassicae* Infection?

**FIGURE 3:**
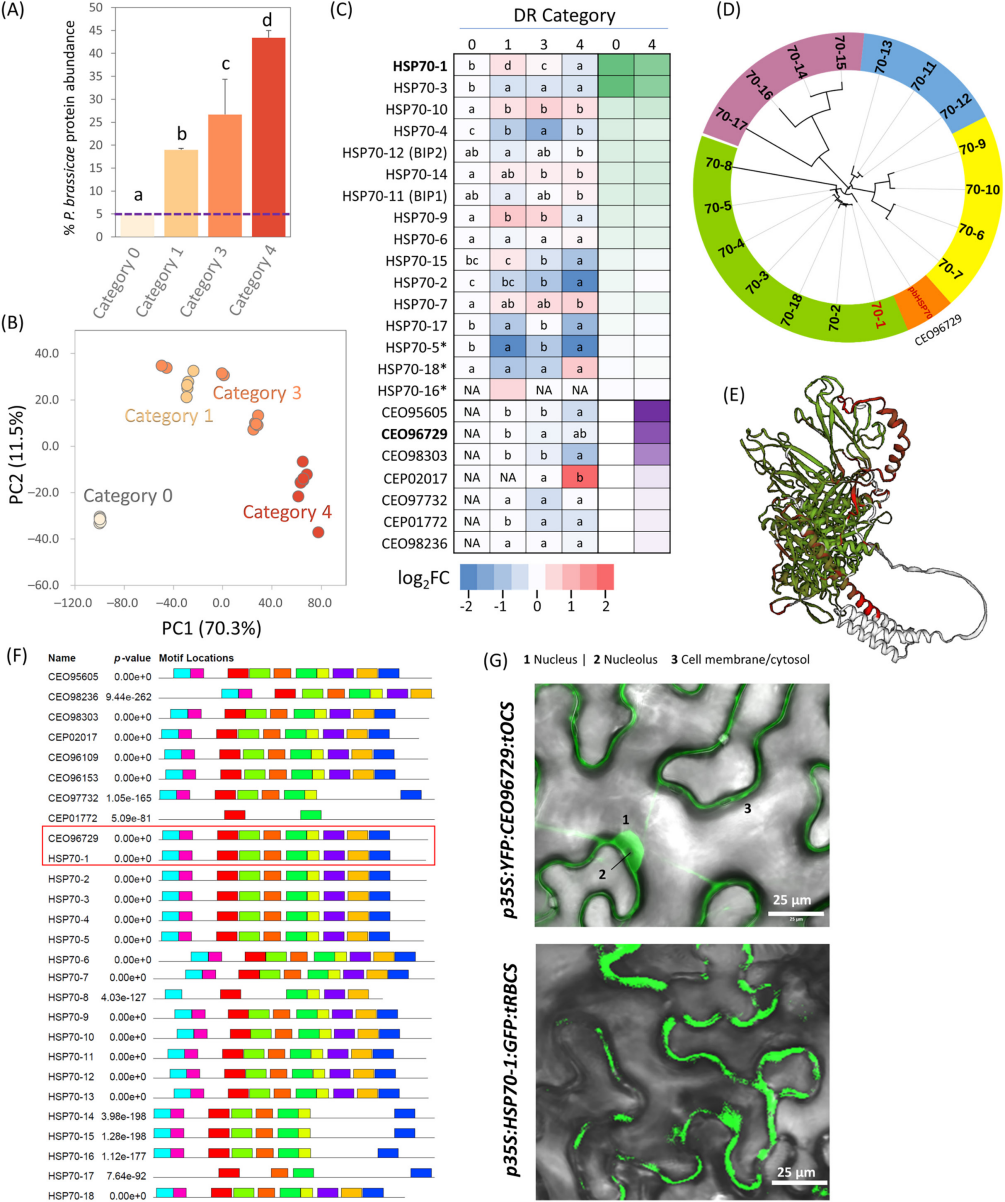
*Arabidopsis* HSP70‐1 shares a high similarity and expected localization with a highly abundant *P. brassicae* HSP70 CEO96729. (A) Estimated *P. brassicae* load in collected samples (based on at least five biological replicates); (B) proteome profile separation based on abundances of 2681 *Arabidopsis* and 1103 *P. brassicae* proteins (*n* ≥ 5); (C) estimated protein content of HSP70 family proteins based on three most abundant unique peptides. Relative log_2_ fold change (blue to red gradient) and composition of HSP70 family in *Arabidopsis* (green) and *P. brassicae* (purple). Statistical significance was determined using the Kruskal–Wallis and Conover tests (*p* < 0.05; *n* ≥ 5). DR Categories (0 = no symptoms; 1 = minor swellings; 2 = thickened primary and lateral roots; 3 = reduced root system with visible galls; 4 = single, large gall). Asterisks indicate proteins with less reliable quantitative data based on fewer than three unique peptides (Table S5). For heatmap visualization, protein abundance values were normalized relative to the abundance observed in category 0; (D) phylogenetic analysis and (E) alignment of CEO96729 and HSP70‐1 protein structures created using SWISS‐MODEL (swissmodel.expasy.org; Waterhouse et al. 2018). White, no alignment; red—low consistency; green, high consistency; (F) conserved motifs of HSP70 family in *Arabidopsis thaliana* and *P. brassicae* ; (G) localization of HSP70‐1 and CEO96729 determined using *Agrobacterium‐*mediated transient transformation of *Nicotiana benthamiana*. Representative images based on three biological replicates.

The pathogen load estimation revealed a surprisingly high abundance of false‐positive *Plasmodiophora* proteins identified in control root tissues. Upon closer inspection, we found that a major protein contributing to these incorrect assignments was CEO96729 (ranking in the top 10 *P. brassicae* proteins and representing some 2% of its proteome), which shares over 70% sequence identity with HSP70‐1 and exhibits shared tryptic peptides. This coincidence, coupled with the observation that HSP70‐1 accumulates in a more resilient *hsp70‐5* mutant (Figure 2A) and its absence promotes infection progression (Figure 1E), piqued our interest. Consequently, we decided to investigate the detectable HSP70 proteins in detail. We collected gall tissues from Col‐0 genotype and used the three most abundant unique peptides to estimate HSP70 protein abundances throughout the gall development process (Figure 3A–C). Despite the limitations inherent to bottom‐up proteomics, which may underestimate post‐translationally modified peptides, our analysis confirmed the dominance of HSP70‐1 in the *Arabidopsis thaliana* root proteome, comprising approximately 25% of total HSP70 isoforms. Its abundance transiently increased following *P. brassicae* infection, subsequently declining to 85% of control levels in fully developed galls (Category 4; Figure 3A). HSP70‐5, representing less than 1% of total HSP70 family abundance, further decreased upon infection. The *P. brassicae* HSP70 family, comprising seven detectable members in the gall proteome, was dominated by CEO95605 (putative ortholog of *Arabidopsis* BIP proteins HSP70‐11,12, and 13; 65%–66% identity) and CEO96729, each accounting for approximately 31% of total HSP70 family abundance in developed galls. Sequence analysis (Figure 3D), 3D structural overlap (Figure 3E), and motif analysis (Figure 3F) showed that these proteins could potentially compete for substrates of the host HSP70s or form heterodimers if colocalized.

To assess this possibility, we performed confocal microscopy on *Nicotiana benthamiana* transiently transformed with *Agrobacterium tumefaciens* carrying *35S* promoter‐driven GFP/YFP‐tagged HSP70 proteins. We observed similar localization patterns for HSP70‐1 and CEO96729, with proteins observed in nuclei, cytosol, or potentially associated with cell membranes (Figure 3G).

### 3.7 CEO96729 Interacts With Host Proteins and Forms Heterodimers With Plant HSP70 Isoforms

**FIGURE 4:**
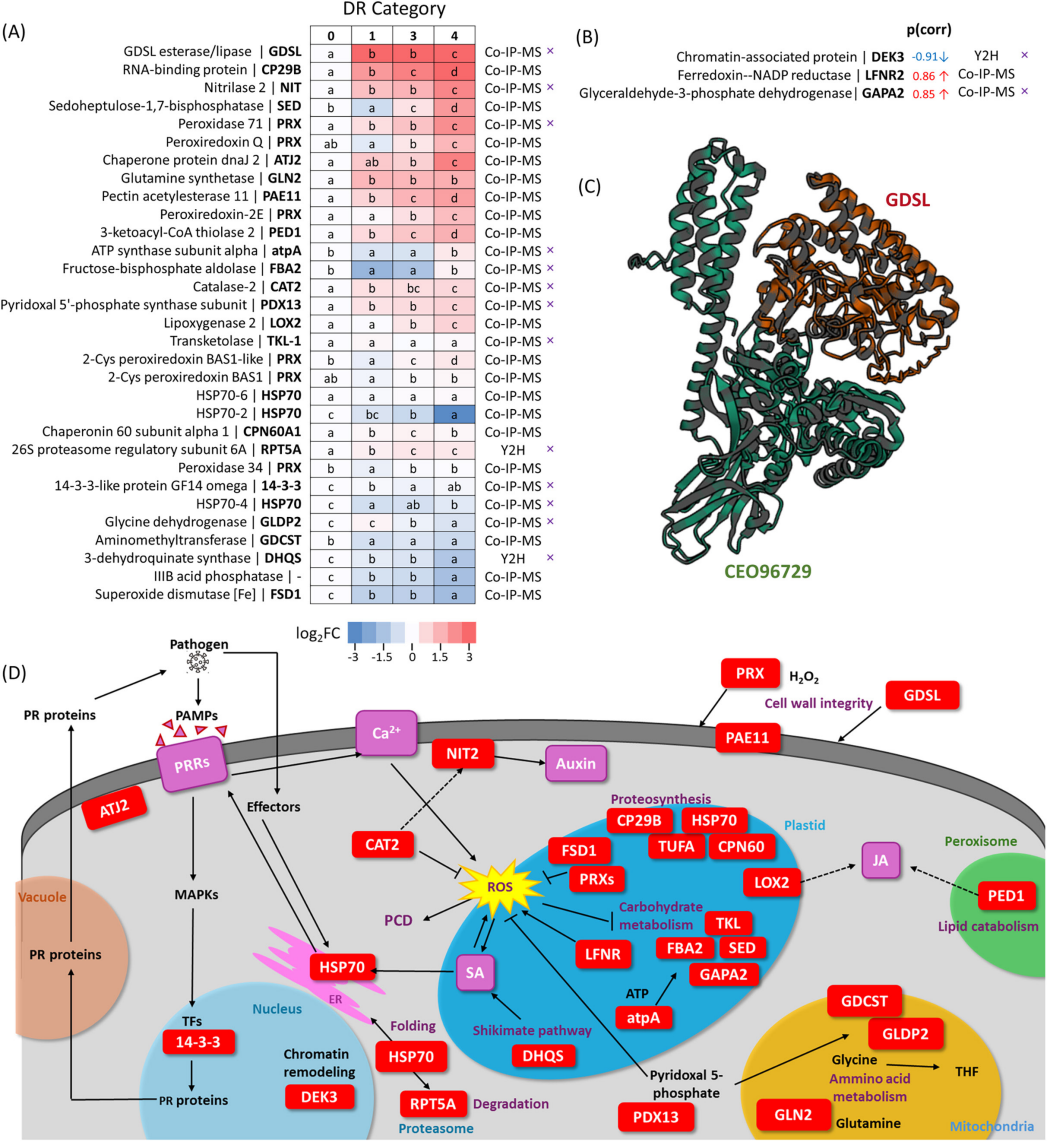
Putative interactors of CEO96729 have a role in response to *P. brassicae* . (A, B) Response of *Arabidopsis* candidate ce096729 interactors to *P. brassicae* infection. Selected proteins identified through co‐immunoprecipitation coupled with mass spectrometry (Co‐IP‐MS) or yeast two‐hybrid (Y2H) screening were analyzed for changes in abundance in response to *P. brassicae* (Table S5). Data are visualized as a heatmap (A), with statistical significance determined using the Kruskal–Wallis test followed by Conover's post hoc analysis (*p* < 0.05, *n* ≥ 5). DR Categories (0 = no symptoms; 1 = minor swellings; 2 = thickened primary and lateral roots; 3 = reduced root system with visible galls; 4 = single, large gall). For heatmap visualization, protein abundance values were normalized relative to the abundance observed in category 0; OPLS/VIP results (B) summarize correlations with infection response for proteins not identified in the validation experiment but found in screening of mutant genotypes (Table S3). Predicted HSP70 interactors are marked with a cross (×). (C) Putative heterodimeric structure of ce096729 with GDSL protein. The structure was predicted using AlphaFold2 through the Neurosnap (Neurosnap Inc., 2022; https://neurosnap.ai/) and visualized with Mol* Viewer (Sehnal et al. 2021); (D) schematic representation of the putative roles and subcellular localizations of CEO96729 interactors. For details, refer to Tables S3, S5 and S6.

The high sequence similarity between CEO96729 and plant HSP70‐1 aligns with the evolutionary conservation typical of HSP70 proteins. However, the observed relationship between CEO96729 abundance and pathogen load, coupled with the compromised resilience of the *hsp70‐1* mutant, suggests that CEO96729 may contribute directly to pathogenic processes. To test this, we conducted yeast two‐hybrid (Y2H) library screening and co‐immunoprecipitation followed by mass spectrometry (Co‐IP‐MS) analysis to identify putative CEO96729 interactors (Figure 4; Table S6). Besides *N. benthamiana*, *Brassica napus* was also used for transient *HSP70* expression and Co‐IP‐MS experiments. Given HSP70's integral role in protein homeostasis, a broad spectrum of interactors was anticipated. To prioritize biologically relevant targets, we compared CEO96729 and HSP70‐1 interactomes, excluding proteins also detected in *35S:GFP* controls. This refined analysis revealed 95 candidate interactors (Table S6), including multiple HSP70 isoforms, supporting CEO96729's capacity to form heterodimers with host HSP70 proteins. Among the 95 candidate interactors, 31 were predicted to associate with HSP70 proteins.

### 3.8 Putative CEO96729 Interactors Have a Role in *P. brassicae* Infection

To further investigate their roles, we quantified the abundance of these putative interactors across distinct categories of gall development. This analysis aimed to delineate dynamic changes in their abundance patterns, shedding light on their potential contributions to CEO96729‐mediated processes and the broader host‐pathogen interaction network. In total, 65 were found in at least one of our datasets, and most of these showed trends consistent with the disease progression. The most interesting candidates are summarized in Figure 4A,B. The identified interactors encompass a range of processes integral to plant‐pathogen interactions, including ROS metabolism, plant immunity, lipid metabolism, carbohydrate metabolism, hormonal signaling, and membrane transport. Notable candidates include orthologs of extracellular GDSL esterase/lipase (AT1G54020), which may modify plant cell wall lipids to facilitate pathogen invasion and gall development by weakening host barriers. Several enzymes implicated in ROS metabolism (Peroxidase 71, AT5G64120; Catalase 2, AT4G35090; Superoxide dismutase, AT4G25100) and ROS signaling (Peroxiredoxins) suggest *Plasmodiophora brassicae* might exploit these proteins to modulate oxidative stress responses and alter cell wall composition during infection. Nitrilase 2 (AT3G44300), associated with auxin biosynthesis, emerges as a potential contributor to gall formation, a hallmark of clubroot disease. Other candidates, such as glutamine synthetase (AT5G35630) and lipoxygenase 2 (AT3G45140), point to metabolic reprogramming and lipid signaling as key pathways targeted by the pathogen. Pyridoxal 5′‐phosphate synthase subunit (AT5G01410), whose mutants display impaired root cell division and elongation, further underscores the pathogen's manipulation of root developmental processes. Additionally, pectin acetylesterase 11 (AT5G45280), capable of modifying cell wall structure, likely facilitates pathogen entry or gall tissue expansion.

### 3.9 HSP70 Is Highly Abundant in Xylem Sap Proteome

**FIGURE 5:**
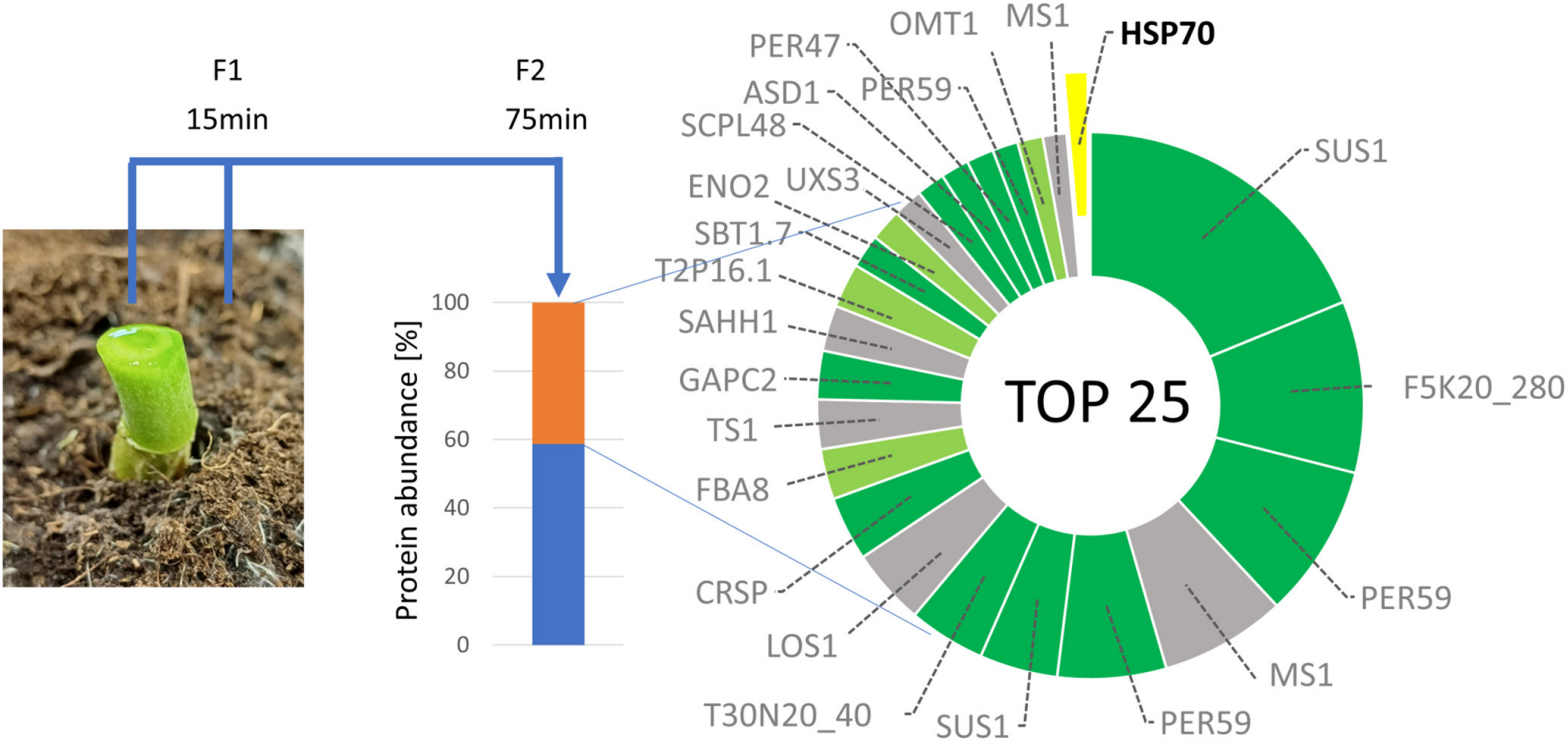
Plant HSP70 proteins are components of *B. napus* xylem sap. Estimated abundances of the 25 most abundant proteins account for more than 40% of the xylem sap proteome. Dark green, known extracellular proteins; lime green, predicted extracellular proteins; gray, not believed to be extracellular. Plants were pretreated with 1 μM of flg22 as described in Kopecká and Černý 2024. The second fraction, collected 15–75 min after dissection and representing a volume of 200–250 μL, was subsequently analyzed. Based on five biological replicates. For details, see Table S7.

Among the top‐scoring interactors identified was an extracellular GDSL esterase/lipase (Figure 4). This observation suggests a potential role for plant HSP70 in the extracellular space, hinting at a novel dimension of interaction between host and pathogen‐derived HSP70s, analogous to mechanisms observed in the animal immune system. However, the localization of plant HSP70 in the extracellular space remains unconfirmed. To address this, we analyzed xylem sap collected from *Brassicae* roots using the methodology recently described by Kopecká and Černý (2024). Plants were pretreated with the elicitor flg22 to mimic biotic interaction. Strikingly, HSP70‐1 was identified among the most abundant proteins in the sap, alongside other well‐characterized extracellular components (Figure 5).

## 4 Discussion

HSPs are indispensable components of cellular stress responses, safeguarding plants against various environmental challenges. However, some pathogens have adapted to exploit these proteins for their own benefit. Emerging evidence suggests that pathogens can hijack HSPs to facilitate infection. For instance, HSP70s play crucial roles in the life cycle of plant viruses, interacting with viral coat proteins to facilitate their nuclear import (Gorovits et al. 2016). Furthermore, cytosolic HSP70 has been identified as an essential component of the plant's hypersensitivity response (Kanzaki et al. 2003) and certain pathogens, such as *Phytophthora infestans* , produce effectors that interact with HSP70 and interfere with its function, promoting cell death and facilitating infection (Lee et al. 2018). Conversely, pathogen‐derived HSP70s can also play significant roles in virulence. For example, bacterial HSPs have been implicated in compromising plant defenses and facilitating the transfer of bacterial DNA into plant cells during infection (Tsai et al. 2012). Additionally, the deletion of a lumenal HSP70 (*FpLhs1*) in *Fusarium pseudograminearum* resulted in reduced growth, conidiation, and pathogenicity (Chen et al. 2019). These findings highlight the multifaceted nature of HSPs, where they can serve both host and pathogen interests in the ongoing arms race between plants and their microbial adversaries. Previous studies have noted the impact of *P. brassicae* on HSP70 regulation, although its significance was not fully recognized at the time (Zhao et al. 2017). In this study, we demonstrate that *Arabidopsis* HSP70 proteins play a crucial role in *P. brassicae* infection. We show that a mutation in *HSP70‐5*, a gene encoding a protein accumulated upon infection (Figure 1A), results in delayed disease onset (Figure 1C–F). Detailed analysis of single mutants in key HSP70 members suggests that this effect may be linked to the interaction between HSP70‐5 and HSP70‐1. Notably, the abundance of HSP70‐1 significantly increases in *hsp70‐5* mutant roots, and *hsp70‐1* mutants exhibit the second‐worst disease impact (Figure 1F). However, this increase in HSP70‐1 alone does not promote resistance, as evidenced by the susceptibility of the *hsp70‐16* mutant (Figure 1F). This observation suggests a functional divergence within the HSP70 family. Prediction algorithms and databases designate HSP70‐5 as exclusively cytosolic, despite some evidence suggesting potential nuclear translocation for all cytosolic HSP70s (Zhao et al. 2021). In contrast, the dual localization (cytosol and nucleus) of HSP70‐1 and HSP70‐16 is well‐established (Berka et al. 2022). This possible differential subcellular partitioning could imply that the extranuclear function of HSP70, potentially involving protein folding, complex assembly, or interaction with cytosolic signaling components, plays a crucial role in the plant's response to *P. brassicae* infection. An alternative hypothesis for the limited impact of HSP70‐1 accumulation on *hsp70‐16* resilience is the involvement of other HSP70 isoforms. Notably, HSP70‐4, HSP70‐12, and HSP70‐17 exhibit significantly different abundances in *hsp70‐5* compared to *hsp70‐16* (Figure 2A). Additionally, the formation of HSP70 heterodimers, which is well‐documented in the literature and also supported by our data, may play a critical role in modulating the functional HSP70 network and thus influence the defense response.

### 4.1 Functional Implications of HSP70 Proteins in *P. brassicae* Pathogenicity and Host–Pathogen Interactions

Interestingly, HSP70‐1 emerged as the most abundant HSP70 protein in our datasets (Figures 1B and 3C), and its abundance in *P. brassicae* ‐infected tissues may have been underestimated due to peptide overlap with the *P. brassicae* HSP70 protein CEO96729. The observed sequence similarity and peptide sharing prompted us to perform a detailed analysis of CEO96729 and other HSP70 proteins in the *P. brassicae* proteome. Within the *P. brassicae* proteome, two major HSP70 proteins accounted for more than 60% of the estimated HSP70 abundance. Among them, only CEO96729 (the putative ortholog of HSP70‐1) exhibited limited fold changes during infection progression (Coefficient of Variation, 5%), in contrast to the BIP ortholog CEO95605 (Coefficient of Variation, 21%). Notably, CEO96729 abundance correlated strongly with pathogen load, suggesting its role in maintaining the *P. brassicae* proteome and potentially contributing directly to pathogenicity. Pathogens utilize their HSP70 proteins to stabilize and fold effector proteins prior to secretion into host cells. Chaperones also play a direct role in secretion mechanisms. For example, bacterial type III secretion systems rely on chaperone‐mediated effector delivery (LeBlanc et al. 2021). In mammalian systems, tumor cells and viruses are known to secrete HSP70‐containing vesicles to influence recipient cells (Mambula et al. 2007; Linder and von Strandmann 2021). These observations raise the possibility of analogous mechanisms in plant‐pathogen interactions.

Initial studies reporting the presence of pathogen HSP70 in the extracellular space attributed this to limited cell lysis, as previously suggested for the secretome of *P. syringae* (Schumacher et al. 2014). However, the discovery of HSP70 within extracellular vesicles released by *Turnip mosaic virus* (Movahed et al. 2019) suggests that its secretion by plant pathogens constitutes an active mechanism in plant‐pathogen interactions. By capturing CEO96729 interactors, we identified an interaction with an extracellular GDSL esterase/lipase. This enzyme has been implicated in the regulation of phloem‐mediated long‐distance signaling, which controls plant responses to both biotic and abiotic stress (Breitenbach et al. 2014). Additionally, various members of the GDSL family are known to play critical roles in plant immunity (Oh et al. 2005; Kim et al. 2013). We observed a substantial accumulation of this protein in response to *P. brassicae* , with one of the highest fold changes in our dataset. This finding provides indirect evidence for a role of CEO96729 in the extracellular space, and the interaction between CEO96729 and GDSL esterase/lipase suggests a potential mechanism by which *P. brassicae* may disrupt systemic plant responses, thereby modulating the host's immune and stress signaling pathways.

However, the striking sequence and structural similarity between CEO96729 and its host homolog, HSP70‐1 (Figure 3D–F), coupled with the results of pathogenicity assays (Figure 1), strongly suggest a more multifaceted mode of action. Notably, plant xylem sap proteomes of *Solanum tuberosum* and *Hordeum vulgare* contain HSP70 proteins (Kopecká and Černý 2024), and the same was found in *B. napus* (Figure 5). Data from the SUBA database further support extracellular localization of HSP70‐1 in *Arabidopsis* (Hooper et al. 2017). These findings suggest that HSP70‐1 and CEO96729 may colocalize and interact within the extracellular space. Indeed, our Co‐IP‐MS experiments captured heterodimeric complexes involving HSP70 and CEO96729 (Figure 4). While HSP70‐1 was also detected in these experiments (Table S6), it was excluded from Figure 4 as it did not meet the criterion of being more abundant in the CEO96729 interactome compared to its own interactome. Its high abundance in the sample is expected, as it served as the bait protein in its own co‐immunoprecipitation.

### 4.2 Identified Putative CEO96729 Interactors Are Known Components of Biotic Stress Response in *Arabidopsis*

While HSP70 has not yet been specifically tracked in entering host cells, its role in stabilizing effectors suggests a potential mechanism for such processes. Notably, the list of putative *P. brassicae* HSP70 interactors identified in this study includes several proteins that appear crucial for the host's response to infection. The proteins within the HSP70 interactome are regulated in response to *P. brassicae* infection (Figure 4) and encompass a broad range of functions relevant to plant‐pathogen interactions. In the context of *P. brassicae* , these proteins likely play pivotal roles in modulating ROS homeostasis, maintaining cellular integrity, adjusting metabolism, modifying cell wall composition, and regulating defense signaling. Oxidative stress‐related proteins, including peroxidases, peroxiredoxins, catalase, and superoxide dismutase, are essential for managing ROS and are central to plant immunity and pathogen resistance. Previous studies have demonstrated that the transduction of HSP70 into cells confers protection against acute oxidative stress induced by H_2_O_2_ (Hino et al. 2023). Furthermore, exogenous application of the HSP70 inhibitor pifthrin increases H_2_O_2_ content and decreases the activity of ascorbate peroxidase 1 (APX1) (Ma et al. 2024). Notably, orthologs of catalase 2 were significantly enriched in both our Co‐IP‐MS experiments, and catalase 2 itself was identified as one of the proteins negatively correlated with pathogen load in the susceptible genotypes *hsp70‐1* and *hsp70‐14* (Table 1). Catalase 2 is pivotal in regulating ROS during pathogen infection and modulating phytohormone signaling. Salicylic acid inhibits catalase 2 activity, leading to elevated H_2_O_2_ levels and subsequent suppression of auxin biosynthesis through posttranslational modification of tryptophan synthetase (Yuan et al. 2017). Interestingly, one of the auxin biosynthetic enzymes, nitrilase 2, was also enriched among the putative CEO96729 interactors. Auxin is crucial for gall formation during *P. brassicae* infection, the indole‐3‐acetonitrile pathway significantly contributes to auxin biosynthesis in *Arabidopsis* roots during clubroot development. Plants transformed with nitrilase in the antisense orientation exhibit delayed clubroot progression, highlighting its functional importance in this context (Neuhaus et al. 2000; Bíbová et al. 2023). The interaction of nitrilase 2 with the pathogen's HSP70 could reflect the requirements of *P. brassicae* for maintaining auxin flow to facilitate gall development. Cell wall‐modifying enzymes, such as pectin acetylesterase, influence pathogen entry and the plant's structural defenses. Metabolic enzymes, including sedoheptulose‐1,7‐bisphosphatase, support carbon, nitrogen, and secondary metabolite pathways often reprogrammed during *P. brassicae* infection. Furthermore, 14–3‐3 proteins, which are frequently targeted by pathogen effectors, play a central role in regulating plant immunity by controlling the activity and function of their binding partners (Sheikh et al. 2024). These proteins have also been shown to bind HSP70 (Hloušková et al. 2019) and were previously reported in response to *P. brassicae* in *Brassica rapa* (Song et al. 2016).

Together, these proteins highlight the interconnected mechanisms that plants utilize to restrict pathogen growth and adapt to biotic stress. It is highly plausible that *P. brassicae* hijacks these pathways, with HSP70 interactions playing a pivotal role in facilitating this process. Our findings do not provide direct evidence of CEO96729 interacting with host proteins during *Plasmodiophora brassicae* infection. However, the combined indirect evidence—demonstrating its specific binding to host proteins in plants overexpressing CEO96729, along with the presence of HSP70 in xylem sap—suggests that CEO96729 may contribute to pathogenesis either through direct interactions with host proteins or indirectly by modulating HSP70 function and disrupting its cellular roles. The abundance of many identified HSP70 interactors increases during infection, which supports this hypothesis and could be interpreted as a cellular response to compensate for the malfunction of these proteins.

## Author Contributions

M.Č., R.K., B.B., S.A., and J.L.M. initiated the study. M.Č., R.K., and S.A. designed the experiments. R.K., S.A., and M.B. cultivated plants, performed inoculations, and collected plant material for analyses. S.A. and J.L.M. conducted disease ratings. R.K., M.L., and D.A. prepared constructs for transient expression, while R.K. and D.A. performed confocal microscopy. M.Č., M.B., and R.K. conducted proteome analyses. R.K. and S.J. contributed to Y2H screening. M.Č., M.B., and R.K. performed co‐IP‐MS. M.Č. and R.K. analyzed data, prepared figures, and wrote the draft manuscript. All authors reviewed the manuscript, and M.Č. finalized the work. All authors approved the published version.

## Supporting information


**Figure S1.** Constructs used for transient transformations.
**Figure S2.** Representative images of plants infected with *P. brassicae* .

**Table S1.** Differentially abundant proteins identified in the roots and shoots of 4‐week‐old *A. thaliana* plants collected 14 days after inoculation. Supporting Information table for Figure 1A.
**Table S2.** Proteins identified in clubroots (Category 4) of Col‐0 and selected mutants in HSP70. Supporting Information table for Figure 2B–D.
**Table S3.** Results of orthogonal partial least‐squares discriminant analysis of proteome profiles. Supporting Information table for Figure 2E–H.
**Table S4.** Manual evaluation of HSP70 family abundances. Supporting Information for Figure 2A.
**Table S5.** Protein abundances across gall development. Supporting Information for Figure 3A–C and Figure 4A.
**Table S6.** Identified interactors. Supporting Information for Figure 4.
**Table S7.** Proteins found in xylem sap proteome. Supporting Information for Figure 5.

## Data Availability

The data that support the findings of this study are available in the Supporting Information of this article, and raw proteomics data were deposited in the ProteomeXchange Consortium (http://proteomecentral.proteomexchange.org) via the PRIDE partner repository with the dataset identifier PXD059131.
