# Supplementary material for: HSP70 as a Mediator of Host–Pathogen Interaction in Arabidopsis thaliana During Plasmodiophora brassicae Infection
Source: Physiol Plant. 2025 Jun 4;177(3):e70309. doi: 10.1111/ppl.70309 (PMC12135032; doi:10.1111/ppl.70309)
Supplement: Supplementary file 1 — Figure S1. Constructs used for transient transformations. Figure S2. Representative images of plants infected with P. brassicae . [file PPL-177-e70309-s001.docx]

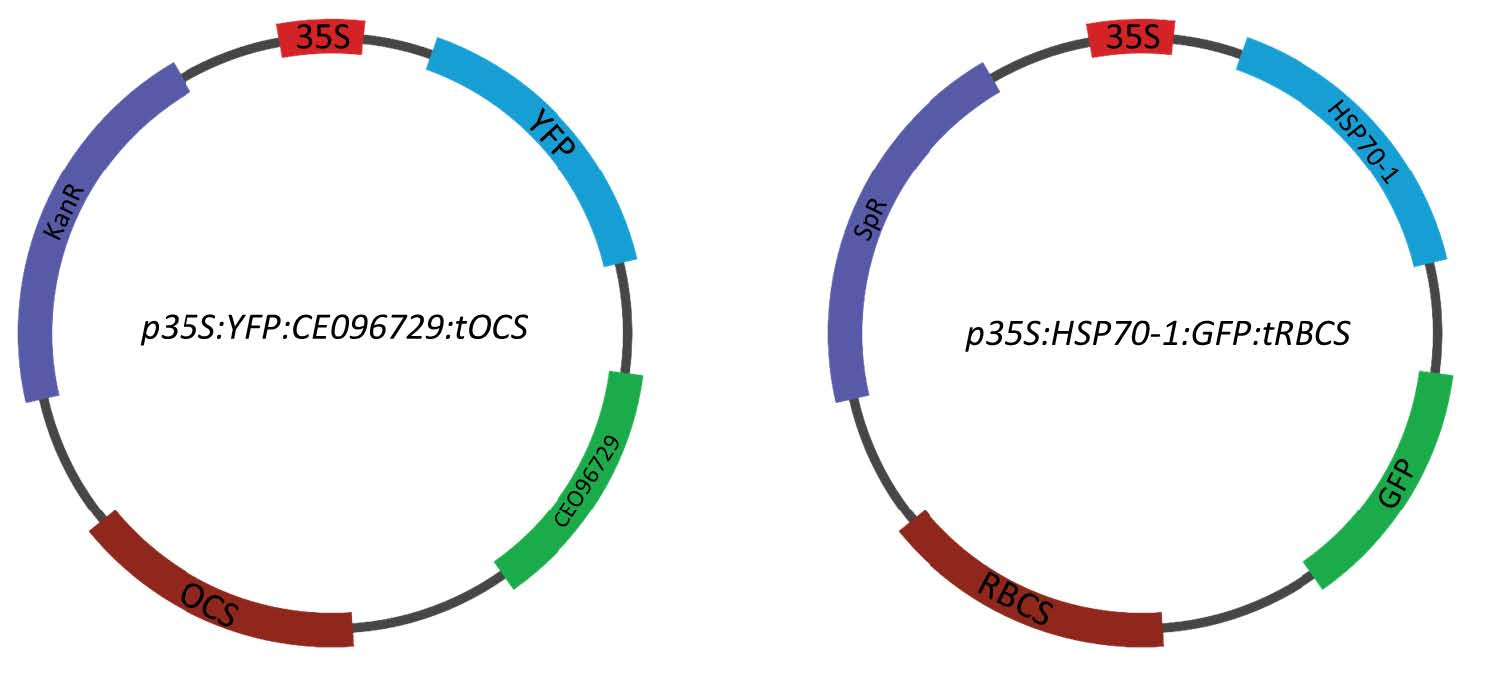


**Supplementary Fig. S1**. The visualization of constructs used for transient expression experiments.

The *HSP70-1* and *CE096729* genes were cloned into expression vectors using Gateway and GreenGate cloning systems, respectively. For *CE096729*, the coding sequence (CDS) was amplified by PCR (GGGGACAAGTTTGTACAAAAAAGCAGGCTTAATGTCTGAGAAGAAAGTCAGTGGC, GGGGACCACTTTGTACA
AGAAAGCTGGGTTTTAGTCGACCTCCTCGATCTTGGG) and cloned into the pDONR207 entry vector by a BP reaction (Gateway BP Clonase II Enzyme mix). Subsequently, the CDS was transferred to the pEarleyGate104 destination vector (Gateway LR Clonase™ II Enzyme mix) to introduce an N-terminal YFP tag. For *HSP70-1*, the CDS was amplified (AGAAGTGAAGCTTGGTCTCAGGCTCCATGTCGGGTAAAGG
AGAAGGA, AGGGCGAGAATTCGGTCTCACTGAGTCGACCTCCTCGATCTTAGG) and cloned into the pGGC000 vector (Addgene plasmid #48858). The GFP tag and linker were cloned into pGGD000 and pGGF000, respectively (Addgene plasmid #48859, #48861). The final construct with a C-terminal GFP tag was assembled using the GreenGate protocol (Addgene, Kit #1000000036) and the pFASTRK destination vector (Decaestecker et al., 2019).


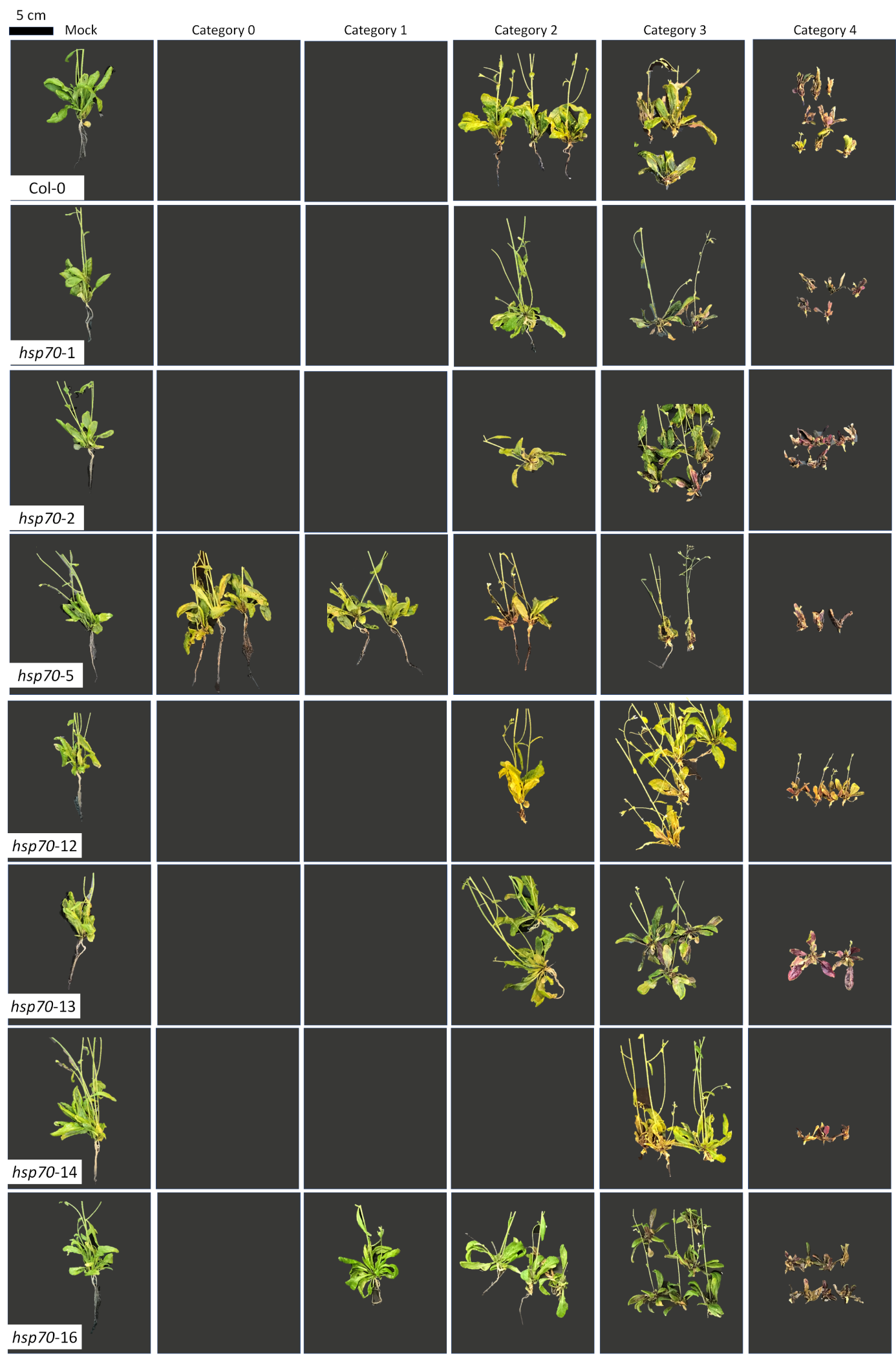


**Supplementary Fig. S2**. Representative images of plants infected with *P. brassicae*. Supplementary figure to Fig. 1. Disease rating (DR) based on root gall development (mock – control, not infected; 1 - minor swellings; 2 - thickened primary and lateral roots; 3 - reduced root system with visible galls; 4 - single, large gall).
